# Differential culprit plaque morphology in acute coronary syndrome: a comparison between very young patients (≤35 years) and older counterparts using optical coherence tomography

**DOI:** 10.1093/ehjimp/qyae046

**Published:** 2024-05-21

**Authors:** Gaurav Chaudhary, Basant Gupta, Shubhajeet Roy, Sharad Chandra, Akhil Sharma, Akshyaya Pradhan, Monika Bhandari, Pravesh Vishwakarma, Rishi Sethi, Sudhanshu Kumar Dwivedi, Vinit Baliyan, Prachi Sharma, Vikash Jaiswal, Abhishek Singh, Ayush Shukla, Sajina Shrestha, Alessia Gimelli

**Affiliations:** Department of Cardiology, King George’s Medical University, Lucknow, Uttar Pradesh, India; Department of Cardiology, King George’s Medical University, Lucknow, Uttar Pradesh, India; King George Medical University, Lucknow, Uttar Pradesh, India; Department of Cardiology, King George’s Medical University, Lucknow, Uttar Pradesh, India; Department of Cardiology, King George’s Medical University, Lucknow, Uttar Pradesh, India; Department of Cardiology, King George’s Medical University, Lucknow, Uttar Pradesh, India; Department of Cardiology, King George’s Medical University, Lucknow, Uttar Pradesh, India; Department of Cardiology, King George’s Medical University, Lucknow, Uttar Pradesh, India; Department of Cardiology, King George’s Medical University, Lucknow, Uttar Pradesh, India; Department of Cardiology, King George’s Medical University, Lucknow, Uttar Pradesh, India; Department of Radiology, Division of Cardiovascular Imaging, Massachusetts General Hospital, Harvard Medical School, Boston, MA 02114, USA; Department of Cardiology, King George’s Medical University, Lucknow, Uttar Pradesh, India; Department of Cardiovascular Research, Larkin Community Hospital, South Miami, FL, USA; Department of Cardiology, King George’s Medical University, Lucknow, Uttar Pradesh, India; Department of Cardiology, King George’s Medical University, Lucknow, Uttar Pradesh, India; Department of Medicine, KIST Medical College, Imadol, Patan, M86M+H4W, Mahalaxmi 44700, Nepal; Department of Imaging, Fondazione Toscana ‘Gabriele Monasterio’, Pisa, Italy

**Keywords:** acute coronary syndrome, intimal thickness, optical coherence tomography, plaque rupture, plaque erosion, thin-cap fibroatheroma

## Abstract

**Aims:**

Underlying mechanisms responsible for acute coronary syndrome (ACS) in young patients compared with older counterparts are yet to be explored with optical coherence tomography (OCT). This study aims to explore underlying mechanisms of ACS in ≤35- (very young) and >35-year-old (older counterparts) ACS patients using OCT.

**Methods and results:**

This was a prospective, single-centre, investigational study. Patients were divided into groups according to age (≤35 and >35 years) and further subdivided according to the underlying mechanism i.e. plaque rupture (PR) and plaque erosion (PE). A total of 93 patients were analysed. Thin-cap fibroatheroma (TCFA) was significantly higher among older counterparts than very young patients for both PR (80.0% vs. 31.8%, *P* = 0.002) and PE (66.7% vs. 6.3%, *P* < 0.001) groups. Microchannels were also significantly more prevalent among older than very young patients for both PR (65.0% vs. 18.2%, *P* = 0.004) and PE groups (55.6% vs.12.5%, *P* = 0.013). Macrophages were significantly higher in older than very young patients for both PR (25.0% vs. 0%, *P* = 0.018) and PE (44.4% vs. 0%, *P* = 0.003) groups. In contrast, fibrous cap thickness was greater in very young than older patients for both PR (105.71 ± 48.02 vs. 58.00 ± 15.76 *µ*m, *P* < 0.001) and PE (126.67 ± 48.22 vs. 54.38 ± 24.21 *µ*m, *P* < 0.001) groups. Intimal thickness was greater in older than very young patients for both PR (728.00 ± 313.92 vs. 342.27 ± 142.02 *µ*m, *P* < 0.001) and PE (672.78 ± 334.57 vs. 295.00 ± 99.60 *µ*m, *P* < 0.001) groups.

**Conclusion:**

Frequency of TCFA, microchannels, macrophages, and intimal thickness was significantly higher in older ACS patients compared with very young patients. However, fibrous cap thickness was significantly greater in very young ACS patients compared with older patients.

## Graphical Abstract

**Graphical abstract:**
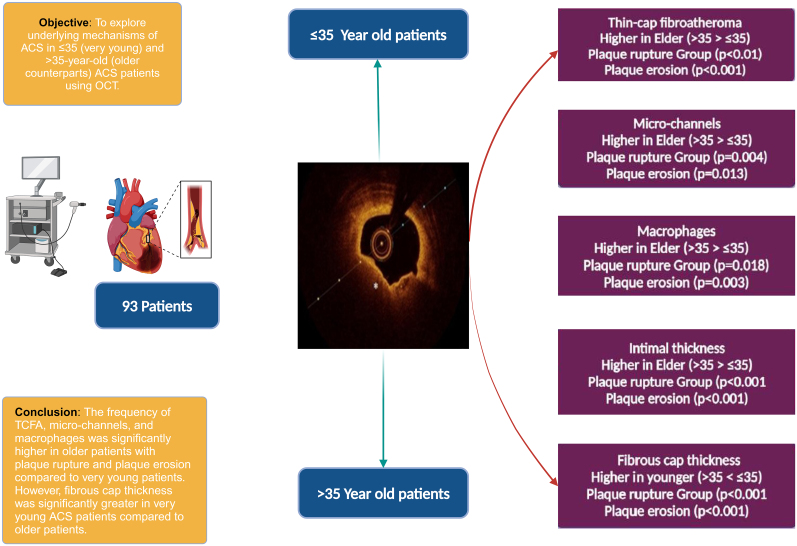
Culprit plaque morphology in acute coronary syndrome patient in very young patients vs. their older counterparts

## Introduction

Acute coronary syndrome (ACS) is a leading cause of morbidity and mortality worldwide with earlier onset in developing countries. Moreover, differing pathophysiology of atherosclerosis in young patients with ACS compared with that of elder patients has been documented.^1,2^

Optical coherence tomography (OCT) is an intravascular imaging tool with resolution of 10–20 *µ*m. Its unparalleled resolution provides unmatched *in vivo* visualization of atherosclerotic plaque microstructure and therefore permits investigation of underlying mechanisms of ACS. Such insights provide details of plaque morphology at various stages of the spectrum ranging from clinically stable to progressive atherosclerotic plaque.^3,4^ Indeed, the benefits of this imaging tool extend far beyond culprit lesion identification and guidance and optimization of interventional procedures and hence validate increasing implementation in routine clinical practice. The present study explored underlying mechanisms of ACS in ≤35- and >35-year-old ACS patients using OCT.

## Methods

### Study design and patient population

This was a prospective, observational, single-centre, investigator-initiated study. The study enrolled all ACS patients that had undergone coronary angiography followed by OCT at a tertiary care centre. Patients were divided into groups according to age [≤35 (very young) and >35 years (older counterparts)] and further subdivided into subgroups according to the underlying mechanism i.e. plaque rupture (PR) and plaque erosion (PE). The study was approved by the Institutional Ethics Committee.

### Inclusion and exclusion criteria

The inclusion criteria was ACS as a trigger event, defined in accordance with the European Society of Cardiology (ESC) guidelines^5^: (i) acute cardiac chest pain or angina equivalent consistent with moderate- to high-risk unstable angina or myocardial infarction lasting more than 10 min duration 72 h before invasive examination, (ii) evidence of ACS requiring catheterization documented by elevated enzymes (>99th percentile or an increase or decrease in creatine kinase myocardial band or high-sensitivity troponin I or T), (iii) electrocardiographic evidence of ST-depression > 1 mm in two or more contiguous leads after the J-point and/or transient ST-elevation > 1 mm in two or more contiguous leads lasting more than 30 min, or (iv) ST-elevated ACS with onset <24 h prior to chest pain >30 min ST-elevation > 1 mm in two or more contiguous leads or new left bundle block. Non-ST-segment elevation myocardial infarction (NSTEMI) was defined as ischaemic symptoms in the absence of ST-segment elevation on electrocardiography with elevated cardiac markers. Unstable angina pectoris was defined as newly developed/accelerating chest pain on rest. The exclusion criteria were: (i) systolic heart failure with left ventricular ejection fraction ≤ 30%; (ii) cardiogenic shock or heart failure requiring intubation, inotropes, diuretics, or mechanical circulation support; (iii) refractory ventricular arrhythmia requiring pharmacologic or defibrillator therapy; (iv) renal insufficiency with serum creatinine: ≥1.5 mg/dL; (v) ACS with culprit lesion in a bypass graft; or (vi) culprit lesions with anatomy unsuitable for OCT evaluation such as severe calcification, extreme tortuosity, distal location, or infarct vessel diameter < 2 or >4 mm.

### Procedures

All patients fulfilling the inclusion criteria were enrolled in the study, in the duration from 01 August 2019 to 30 November 2020. All necessary history was collected from all patients. All patients underwent routine and necessary blood investigations, electrocardiography, echocardiography, and coronary angiography. Coronary angiography was performed on all patients using standard percutaneous techniques. The culprit lesion was identified with coronary angiography, and the results were analysed.

After that OCT was performed with the proximal and distal 5 mm reference segments using frequency–domain OCT imaging system and the Dragonfly catheter (St Jude Medical/Ilumien Optis). A 2.7 F OCT imaging catheter was carefully advanced to distal to the culprit lesion. The automated pullback was performed at 20 mm/s, while blood was displaced by short injection of the contrast media. The images were taken and digitally stored for offline analysis.

### OCT image analysis

**Figure 1:**
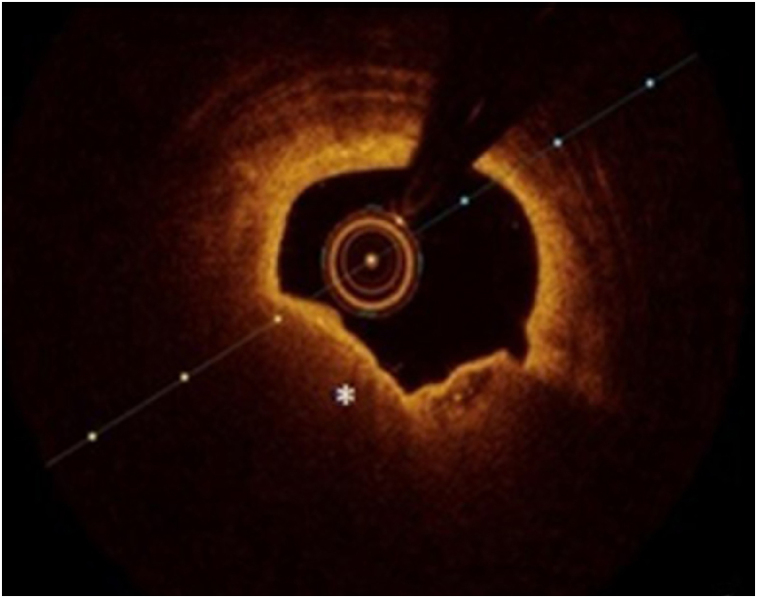
Optical coherence tomography showing fibroatheromatous plaque (star).

**Figure 2:**
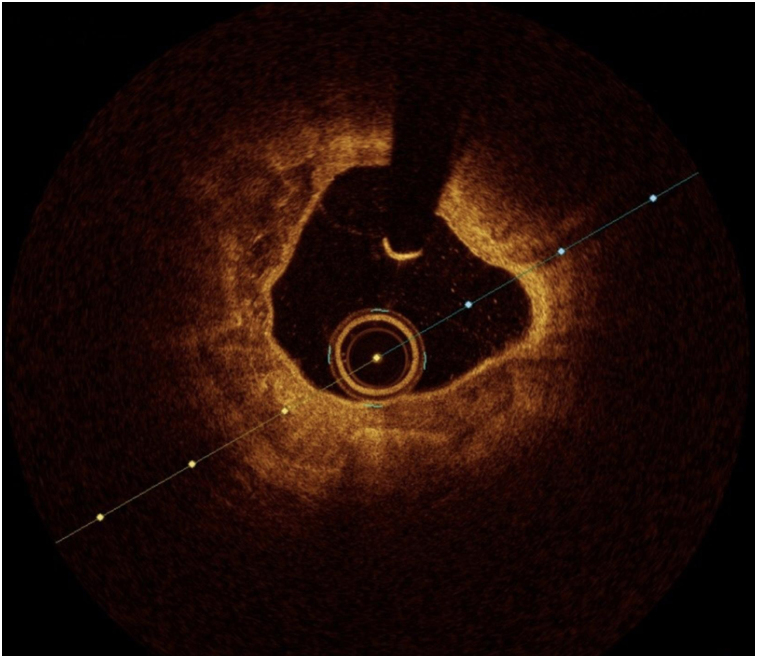
Optical coherence tomography showing fibrocalcific plaque with calcium.

**Figure 3:**
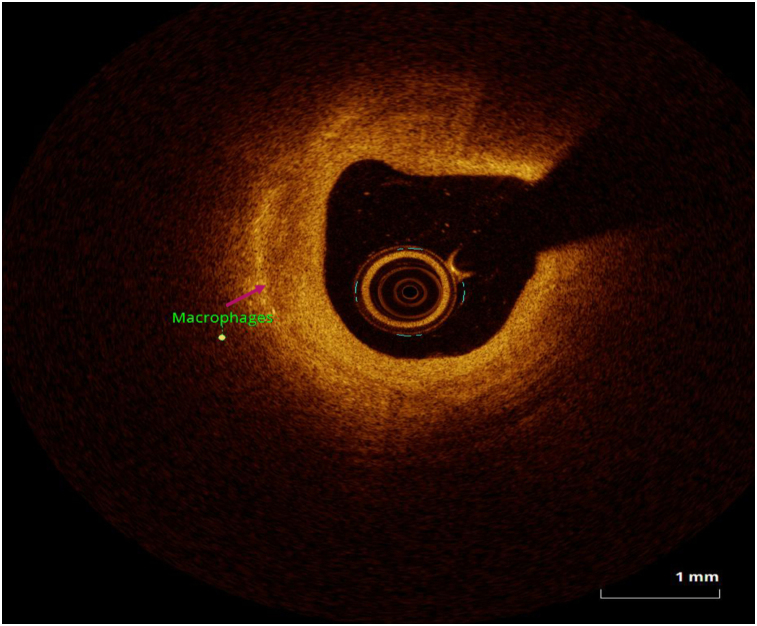
Optical coherence tomography showing macrophages.

**Figure 4:**
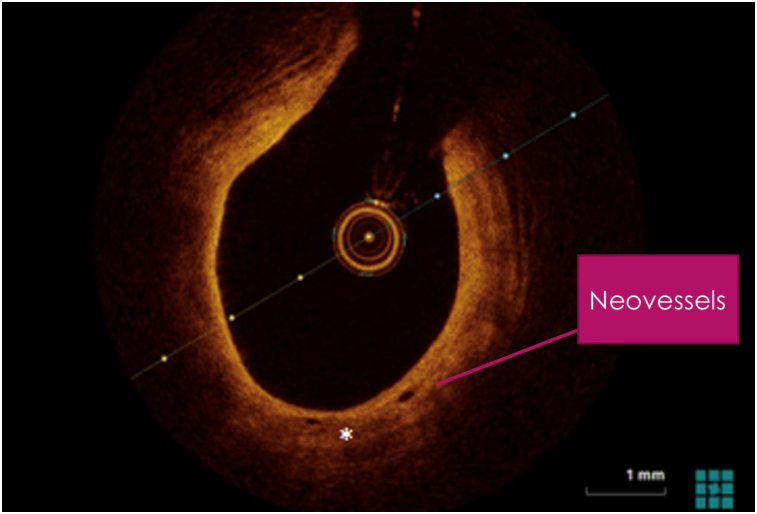
Optical coherence tomography showing neovessels.

**Figure 5:**
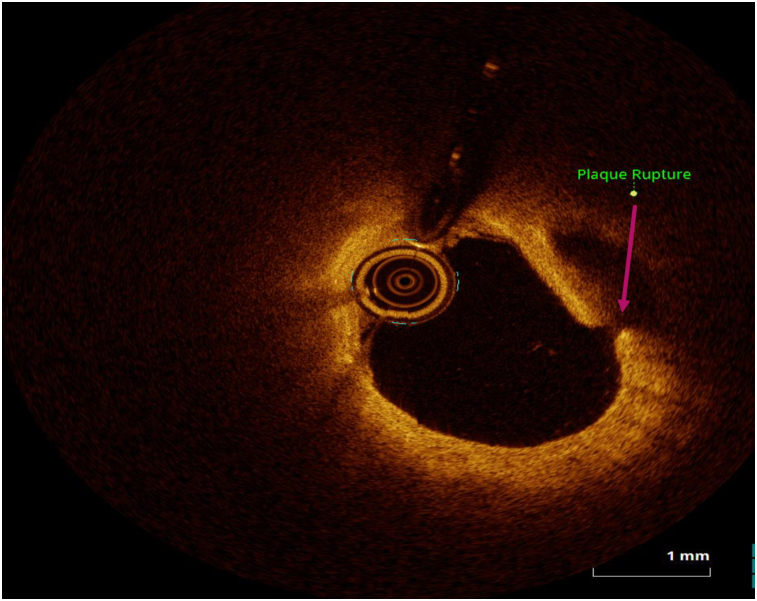
Optical coherence tomography showing plaque rupture.

**Figure 6:**
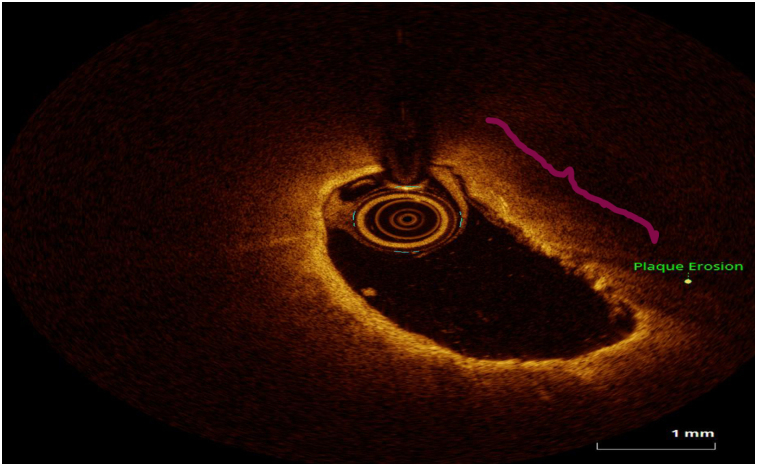
Optical coherence tomography showing plaque erosion.

**Figure 7:**
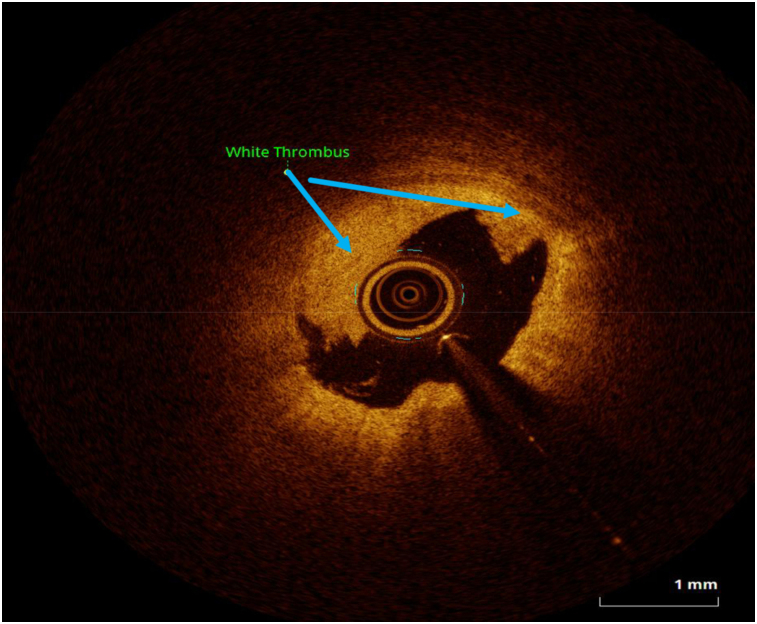
Optical coherence tomography showing white thrombus.

**Figure 8:**
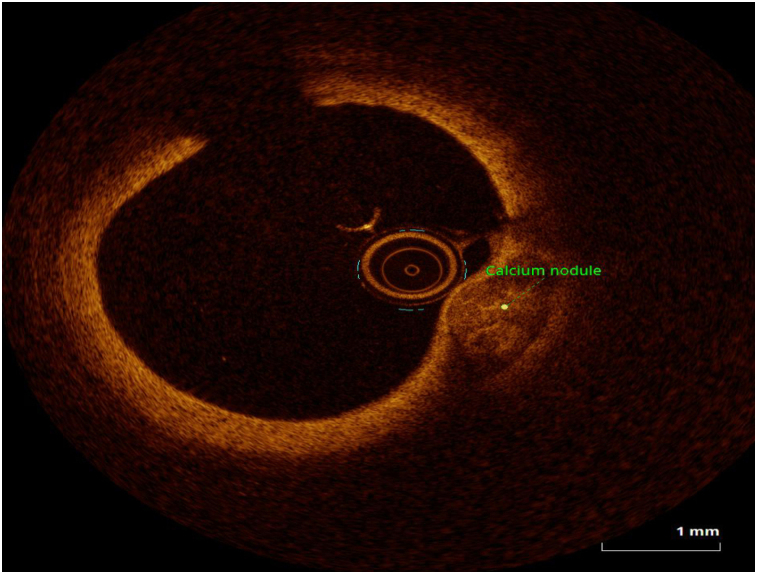
Optical coherence tomography showing calcium nodule.

**Figure 9:**
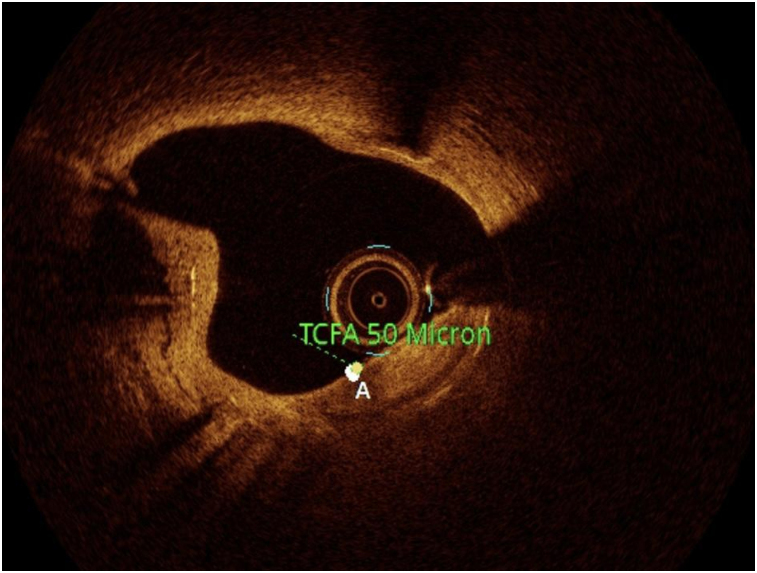
Optical coherence tomography showing TCFA.

OCT image analysis was performed offline by an experienced investigator. Fibrous plaque was identified as a homogeneous, signal-rich area, overlying a lipid core.^6–8^ Fibroatheroma (lipid-rich) plaque was identified as plaque with a fibrous cap thickness < 400 *µ*m over a lipid core extending for >90° in the 10 consecutive frames^6–8^ (*Figure 1*). Fibrocalcific plaque was identified by evidence of fibrous tissue along with calcium^6–8^ (*Figure 2*). Calcium was identified as a signal-poor region with sharply delineated borders.^6,7^ Macrophages were defined as signal-rich, distinct, or confluent punctuate regions that exceeded the intensity of background speckle noise^6–8^ (*Figure 3*). Neovessels were no signal tubular luminal structures without connection to the vessel lumen, recognized on three consecutive cross-sectional images^8^ (*Figure 4*). Plaque rupture was identified by the presence of fibrous cap discontinuity and cavity formation into plaque that communicates with the lumen^6–8^ (*Figure 5*). Plaque erosions were defined as the presence of intracoronary thrombus over the luminal surface of plaque in the absence of fibrous cap discontinuity and without cavity formation^6–8^ (*Figure 6*). Thrombus was considered as an irregular mass protruding into the lumen (mural thrombus) or a luminal mass that is not connected to the vessel wall^8^ (*Figure 7*). Calcific nodules were single or multiple regions of calcium that protruded into the lumen, frequently with sharp angles^6–8^ (*Figure 8*). In the case of ruptured plaques, fibrous cap thickness was assessed both at the non-ruptured site and rupture site.^6–8^ Thin-cap fibroatheroma (TCFA) was defined as the thinnest fibrous cap ≤65 *μ*m^6–8^ (*Figure 9*). For assessment of lesion severity, minimum luminal area (MLA) was estimated as the cross-section with the smallest lumen area.^6,7^

### Statistical analysis

Categorical variables are presented as counts and percentages and were compared using the *χ*^2^ test or Fisher’s exact test. Continuous variables are presented as mean ± standard deviation and were compared using the independent samples *t*-test for two groups or analysis of variance (ANOVA) for three or more group comparisons followed by a *post-hoc* test. The data were analysed using the Statistical Package for Social Sciences (SPSS, Chicago, IL, USA) software version 26. A *P* value < 0.05 was considered significant, and all *P* values are two-sided.

## Results

**Figure 10:**
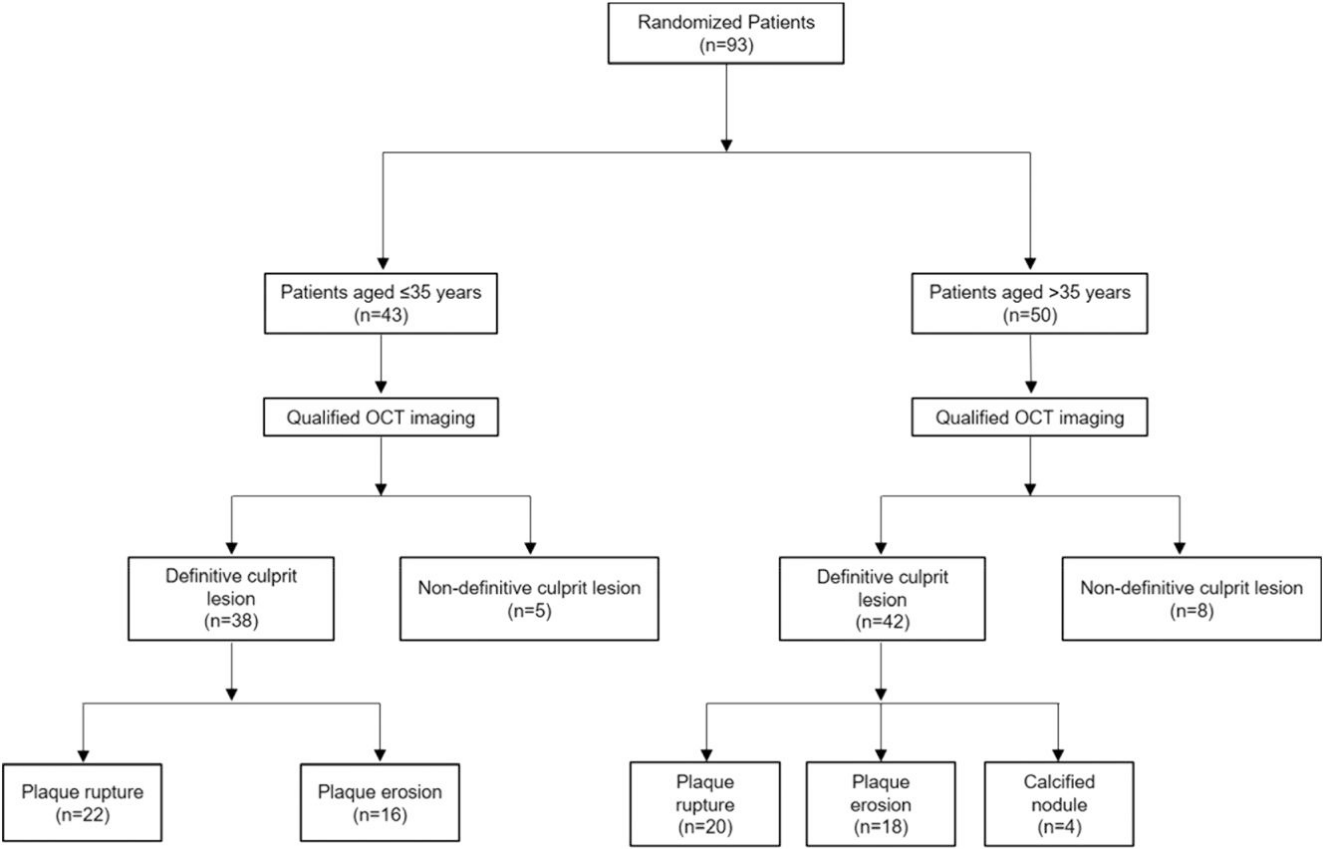
Study flow.

A total of 93 patients were included in the present analysis. Of these 93 patients, 43 patients were ≤35 years (very young) and 50 patients were >35 years (older counterparts). Of the 43 very young patients, 38 had definite culprit lesions. Of these lesions, plaque rupture was identified in 22 patients while plaque erosion was identified in 16 patients. Of the 50 older patients, 42 definite culprit lesions were identified. Of these lesions, plaque rupture, plaque erosion, and calcified nodules were identified in 20, 18, and 4 patients, respectively. The study flow is illustrated in *Figure 10*.

### Baseline characteristics and angiographic findings

Comorbidities such as diabetes and hypertension differed significantly according to age and underlying mechanisms. Diabetes mellitus was significantly more prevalent among older than very young patients (32.0% vs. 4.7%, *P* = 0.001). Similarly, hypertension was significantly more prevalent among older than very young patients (50.0% vs. 4.7%, *P* < 0.001). Unstable angina was significantly more prevalent among older patients than very young patients (18.0% vs. 2.3%, *P* < 0.002). Single-vessel disease was more prevalent among very young patients than older patients, while double-vessel and triple-vessel diseases were more prevalent in the older than very young patients (36.0% vs. 14.0%, *P* = 0.018, and 18.0% vs. 2.3% *P* = 0.018). The demographic, risk factor, laboratory, and clinical details of both very young and older patients are detailed in *Table 1*. No significant differences were observed in disease severity, culprit coronary artery, degree of stenosis, or lesion length. The angiographic findings are elaborated in *Table 2*. No significant relationship was found between the risk factors and OCT data.

**Table 1:** Demographics and clinical presentation

Variables	≤35 years	>35 years	*P* value
Age (years)	31.40 ± 3.332	54.68 ± 9.797	<0.001
Male	42 (97.7)	44 (88.0)	0.118
Female	1 (2.3)	6 (12.0)	0.118
Smoking	23 (53.5)	20 (40.0)	0.216
Tobacco	17 (39.5)	17 (34.0)	0.667
Diabetes mellitus	2 (4.7)	16 (32)	0.001
Hypertension	2 (4.7)	25 (50.0)	<0.001
Family history of coronary artery disease	2 (4.7)	3 (6.0)	1.000
Dyslipidaemia	9 (20.9)	15 (30.0)	0.351
ST-elevation myocardial infarction	31 (72.1)	26 (46.0)	0.057
Non-ST-elevation myocardial infarction	11 (25.6)	12 (36.0)	1.000
Unstable angina	1 (2.3)	12 (18.0)	0.002
Single-vessel disease	36 (83.7)	23 (46.0)	<0.001
Double-vessel disease	6 (14.0)	18 (36.0)	0.018
Triple-vessel disease	1 (2.3)	9 (18.0)	0.018

Values expressed as number (%) or mean ± standard deviation.

**Table 2:** Angiographic findings

Variables	OCT-PR (*n* = 42)		*P* value	OCT-PE (*n* = 34)		*P* value
	≤35 years (*n* = 22)	>35 years (*n* = 20)		≤35 years (*n* = 16)	>35 years (*n* = 18)	
Disease severity						
Single-vessel disease	17 (77.3)	10 (50.0)	0.105	14 (87.5)	10 (55.6)	0.102
Double-vessel disease	4 (18.2)	5 (25.0)		2 (12.5)	6 (33.3)	
Triple-vessel disease	1 (4.5)	5 (25.0)		0 (0.0)	2 (11.1)	
Culprit coronary artery						
Left anterior descending artery	17 (77.3)	12 (60.0)	0.349	14 (87.5)	14 (77.8)	0.642
Left circumflex artery	0 (0.0)	1 (5.0)		1 (6.3)	1 (5.6)	
Right coronary artery	5 (22.7)	7 (35.0)		1 (6.3)	3 (16.7)	
Degree of stenosis (%)	81.32 ± 14.10	77.00 ± 17.20	0.377	73.57 ± 15.50	81.11 ± 15.30	0.179
Lesion length (mm)	20.864 ± 7.91	22.650 ± 7.32	0.453	18.563 ± 7.60	17.828 ± 8.04	0.787

Values expressed as number (%) or mean ± standard deviation.

### OCT imaging

TCFA, microchannels, macrophages, and intimal cap thickness (ICT) were significantly higher among older than very young patients for both PR (TCFA: 80.0% vs. 31.8%, *P* = 0.002; microchannels: 65.0% vs. 18.2%, *P* = 0.004; macrophages: 25.0% vs. 0%, *P* = 0.018; and ICT: 728.00 ± 313.92 vs. 342.27 ± 142.02 *µ*m, *P* < 0.001) and PE (TCFA: 66.7% vs. 6.3%, *P* < 0.001; microchannels: 55.6% vs.12.5%, *P* = 0.013; macrophages: 44.4% vs. 0%, *P* = 0.003; and ICT: 672.78 ± 334.57 vs. 295.00 ± 99.60 *µ*m, *P* < 0.001) groups. In contrast, fibrous cap thickness was greater in very young than older patients for both PR (105.71 ± 48.02 vs. 58.00 ± 15.76 *µ*m, *P* < 0.001) and PE (126.67 ± 48.22 vs. 54.38 ± 24.21 *µ*m, *P* < 0.001) groups. Plaque type, thrombus formation, and minimal luminal diameter did not differ significantly according to age for either of the underlying mechanisms. The OCT imaging findings are outlined in *Table 3*.

**Table 3:** OCT imaging findings

Variables	OCT-PR (*n* = 42)		*P* value	OCT-PE (*n* = 34)		*P* value
	≤35 years (*n* = 22)	>35 years (*n* = 20)		≤35 years (*n* = 16)	>35 years (*n* = 18)	
Plaque characteristics						
Plaque type						
Fibroatheroma	21 (95.5)	20 (100.0)	1.000	10 (62.5)	16 (88.9)	0.110
Fibrous	1 (4.5)	0 (0.0)		6 (37.5)	2 (11.1)	
Fibrocalcific	0 (0.0)	0 (0.0)		0 (0.0)	0 (0.0)	
Nil	0 (0.0)	0 (0.0)		2 (12.5)	0 (0.0)	
Thin-cap fibroatheroma	7 (31.8)	16 (80.0)	**0.002**	1 (6.3)	12 (66.7)	**<0**.**001**
Microchannels	4 (18.2)	13 (65.0)	**0.004**	2 (12.5)	10 (55.6)	**0**.**013**
Macrophages	0 (0.0)	5 (25.0)	**0.018**	0 (0.0)	8 (44.4)	**0**.**003**
Cholesterol crystals	4 (18.2)	5 (25.0)	0.714	1 (6.3)	5 (27.8)	0.180
Thrombus						
Red	8 (36.4)	6 (30.0)	0.839	4 (25.0)	2 (11.1)	0.695
White	4 (18.2)	6 (30.0)		5 (31.3)	5 (27.8)	
Mix	9 (40.9)	7 (35.0)		2 (12.5)	3 (16.7)	
Nil	1 (4.5)	1 (5.0)		5 (31.3)	8 (44.4)	
Fibrous cap thickness (*µ*m)	105.71 ± 48.02	58.00 ± 15.76	**<0.001**	126.67 ± 48.22	54.38 ± 24.21	**0**.**002**
Intimal thickness (*µ*m)	342.27 ± 142.02	728.00 ± 313.92	**<0.001**	295.00 ± 99.60	672.78 ± 334.57	**<0**.**001**
Minimum lumen area (mm^2^)	1.97 ± 1.43	1.92 ± 1.13	0.905	2.644 ± 1.78	1.94 ± 0.93	0.170

Values expressed as number (%) or mean ± standard deviation.

## Discussion

Culprit plaque morphology in very young ACS patients differs to that of older patients. These differences are yet to be delineated. The PROSPECT study^9^ analysed *in vivo* coronary plaque characteristics and composition in ACS patients <65 and ≥65 years of age using virtual histology intravascular ultrasound (VH-IVUS). However, the cut-off age of 65 years eluded the possibility of identifying early changes in plaque composition. Moreover, and more importantly, non-culprit rather than culprit lesions were analysed in patients undergoing treatment for only one or two diseased epicardial vessels. The TOTAL-OCT study^10^ compared stenosis severity and plaque content in culprit lesions with intact fibrous cap and plaque rupture using OCT. The OCT-FORMIDABLE registry^11^ investigated culprit plaque characteristics in ACS patients ≤50 and >50 years of age. Yet again, the cut-off age used to divide patients seemed high considering early onset of ACS in recent times. Furthermore, the study did not explore comparison of underlying mechanisms of ACS between the age groups. Neither profile nor the correlations between lipid profile and plaque characteristics was investigated, despite reports as common risk factors in young ACS patients. The present study sought to address this knowledge gap and to the authors’ knowledge, which is the first OCT study to evaluate culprit plaque morphology according to underlying mechanism in ACS patients aged ≤35 years and >35 years. This study also assessed lipid profiles of the included patients.

The present study reports a few major findings: (i) frequency of TCFA among older and very young patients for both PR and PE (80.0% vs. 31.8%, *P* = 0.002, and 66.7% vs. 6.3%, *P* < 0.001), microchannels (65.0% vs. 18.2%, *P* = 0.004, and 55.6% vs.12.5%, *P* = 0.013), and macrophages (25.0% vs. 0%, *P* = 0.018, and 44.4% vs. 0%, *P* = 0.003) was significantly higher in older patients compared with very young patients irrespective of the underlying mechanism; (ii) fibrous cap thickness among very young and older patients for both PR and PE (105.71 ± 48.02 vs. 58.00 ± 15.76 *µ*m and 126.67 ± 48.22 vs. 54.38 ± 24.21 *µ*m, *P* < 0.001) was significantly higher in very young patients compared with older patients irrespective of underlying mechanism; and (iii) intimal thickness of very young and older patients for both PR (342.27 ± 142.02 vs. 728.00 ± 313.92 *µ*m, *P* < 0.001) and PE (295.00 ± 99.60 vs. 672.78 ± 334.57 *µ*m, *P* < 0.001) was significantly lower in very young patients compared with older patients irrespective of the underlying mechanism.

Plaque rupture is distinctive of a disrupted thin fibrous cap overlying a large necrotic core, massive macrophage infiltration, less smooth muscle cells, and expansive remodelling. In contrast, plaque erosion is distinctive of more fibrous tissue, intact fibrous cap, less or deep-seated necrotic core, devoid of endothelial cells, less macrophage infiltration, more smooth muscle cells, and a hyaluronan-rich endothelial matrix.^12^ In earlier times, plaque rupture was believed to be an age-related pathophysiologic mechanism, declining with age. This mechanism was also believed to be the most frequent underlying mechanism for ACS responsible for majority of ST-segment myocardial infarctions, whereas plaque erosion was the second most prevalent underlying mechanism for ACS. However, recent evidence has questioned these beliefs. Kim *et al.*^1^ have demonstrated a trend of increased plaque rupture prevalence that declined only after the age of 80 years. Furthermore, plaque rupture is steadily increasing in prevalence as time advances as reported by earlier studies.^10,13–15^

### Study limitations

The present study was limited by the small sample size. It also did not explore the gender-related differences; however, earlier investigators have already been conducted to assess the same.^2,16^

## Conclusion

The frequency of TCFA, microchannels, and macrophages was significantly higher in older patients with plaque rupture and plaque erosion compared with very young patients. However, fibrous cap thickness was significantly greater in very young ACS patients compared with older patients. These results were attained with the use of high resolution of OCT, which enables high resolution of the microstructure of coronary plaque *in vivo.* This new technique may provide an opportunity to detect earliest high-risk culprit lesion in elder age.

**Disclosure:** The abstract of this study was presented and won best abstract award at the American Heart Association conference (https://www.ahajournals.org/doi/10.1161/circ.144.suppl_1.9935).

## Consent

All patients provided written informed consent prior to study commencement.

## Data Availability

The data underlying this article are available in the article and will be made available upon request from the lead author or corresponding author.

## Lead author biography

Prof. Gaurav Chaudhary, MBBS, MD, DM, FACC, FESC, FSCAI, FAPSIC, is a Professor of Cardiology at the Department of Cardiology, King George’s Medical University, an Editor and EC Member of the UPCSI, and an Associate Editor of *IHJ Cardiovascular Case Reports*. His areas of interest include coronary imaging, optical coherence tomography, rotablation, and structural heart interventions. He has indexed 23 papers and published 20 abstracts and 9 book chapters. He also has attended the following conferences: ACC (March 2019, New Orleans, USA; presented a paper and received an FACC fellowship), UPCSI (February 2019 as Faculty), and NIC (2019 at Lucknow). He is a member of the following societies: FACC (Fellow American College of Cardiology), FESC (Fellow American College of Cardiology), FSCAI, Life Member Cardiological Society of India, Life Member Indian Association of Echocardiography, and Life Member Indian Society of Cardiology.
